# Genetic Diversity of *Phyllanthus emblica* From Two Different Climate Type Areas

**DOI:** 10.3389/fpls.2020.580812

**Published:** 2020-11-30

**Authors:** Xiongfang Liu, Yongpeng Ma, Youming Wan, Zhenghong Li, Hong Ma

**Affiliations:** ^1^Research Institute of Resources Insects, Chinese Academy of Forestry, Kunming, China; ^2^Yunnan Key Laboratory for Integrative Conservation of Plant Species with Extremely Small Populations, Kunming Institute of Botany, Chinese Academy of Sciences, Kunming, China

**Keywords:** *Phyllanthus emblica*, genetic diversity, genetic structure, population dynamics, precipitation, altitude

## Abstract

*Phyllanthus emblica* L. is a well-known medicinal and edible plant species. Various medicinal compounds in the fruit make it an important medicinal and promising economic material. The plant is widely distributed in Southwestern and Southern China. However, due to massive deforestation and land reclamation as well as deterioration of its natural habitat in recent years, the wild resources of this species have been sharply reduced, and it is rare to see large-scale wild *P. emblica* forests so far. In order to effectively protect and rationally utilize this species, we investigated the genetic diversity, genetic structure, and population dynamics of 260 individuals from 10 populations of *P. emblica* sampled from the dry climate area in Yunnan and wet climate area in Guangxi using 20 polymorphic EST-SSR markers. We found high genetic diversity at the species level (*H*e = 0.796) and within populations (*H*e = 0.792), but low genetic differentiation among populations (*F*_*ST*_ = 0.084). In addition, most genetic variation existed within populations (92.44%) compared with variation among the populations (7.56%). Meanwhile, the NJ tree, STRUCTURE, and hierarchical analysis suggested that the sampled individuals were clustered into two distinct genetic groups. In contrast, the genetic diversity of the dry climate group (*H*e = 0.786, *N*a = 11.790, *I* = 1.962) was higher than that of the wet climate group (*H*e = 0.673, *N*a = 9.060, *I* = 1.555), which might be attributed to the combined effects of altitude, precipitation, and geographic distance. Interestingly, only altitude and precipitation had significant pure effects on the genetic diversity, and the former was slightly stronger. In addition, DIYABC analysis suggested the effective population size of *P. emblica* might have contracted in the beginning of the Last Glacial Maximum. These genetic features provided vital information for the conservation and sustainable development of genetic resources of *P. emblica*, and they also provided new insights and guidelines for ecological restoration and economic development in dry-hot valleys of Yunnan and karst areas in Guangxi.

## Introduction

*Phyllanthus emblica* is a perennial deciduous tree or shrub distributed across tropical and subtropical areas. It contains many medicinal compounds such as vitamin C, flavonoids, emblicol, emblicanin A/B, and is used as a daily medication by many ethnic minorities such as Tibetan group in China; its various therapeutic effects such as hepatoprotective, antimicrobials have also been confirmed in modern medical research (Variya et al., 2016; Chaphalkar et al., 2017). The World Health Organization has listed it as one of the three health-care plants that are widely planted throughout the world (Li and Zhao, 2007). Its seeds contain high contents of unsaturated fatty acids, such as linoleic and linolenic acid, making it an important potential raw industrial material (Choudhary and Grover, 2019). In nature, *P. emblica* is mainly pollinated by wind and bees (Bajpai, 1957), and the long-distance transmission of pollen provides the chances of long-distance genetic migration between different populations (Nakanishi et al., 2020). In nature, *P. emblica* usually depends on seed propagation, and long-distance seed dispersal must depend on certain fruit-eating animals (Li and Zhao, 2007). In China, *P. emblica* is mainly distributed south of the Yangtze River with a large distribution in the east-west direction, from the humid area in the east to the dry-hot valley formed by the wind-burning effect in the west. The Yunnan dry-hot valley is located on the southern edge of the Hengduan Mountains in the Biodiversity Center in Southwest China. It exhibits unique characteristics of dry-hot valleys due to its geographical location and climate: the average annual precipitation is 600–800 mm, and the annual average evaporation is 3–6 times higher than the precipitation. Precipitation is mainly concentrated in the rainy season, whereas the dry season is extremely arid, which is similar to the typical savannah climate (Jin and Ou, 2000). In contrast, the area near the Tropic of Cancer in central Guangxi belongs to the south subtropical monsoon climate zone with abundant rainfall. Although these two climate types are clearly different, *P. emblica* is distributed in both of these areas and has adapted well to their respective habitats during its long-term evolution. However, since the 1980s, the wild genetic resources of *P. emblica* in China have sharply decreased, and its natural habitat has become severely fragmented as a consequence of unreasonably high levels of development and land utilization (Li and Zhao, 2007). Thus, it is highly important to explore the genetic diversity of *P. emblica* to be able to effectively protect this medicinal, economic and industrial plant species along with rationally developing and utilizing it as an important resource.

Rational utilization and protection of a species depends on understanding its distribution, differentiation, and factors influencing its genetic diversity, which is the basis of species’ adaptation to changing environment (Solbrig, 1991). Particularly, for long-lived woody species, genetic diversity not only determines their ability to adapt to the environment but also forms the basis for maintaining long-term stability of forest ecosystems (Hamrick et al., 1992; Ony et al., 2020; Wells et al., 2020). With the increase in genetic diversity within species, their ability to adapt to environmental changes increases, whereas the decrease or disappearance of genetic diversity often results in low adaptability, reproduction, and disease resistance (Hamrick et al., 1992). In general, the decline in population size may cause a decline in genetic diversity through genetic drift and inbreeding (Hamrick et al., 1992). In the long term, the decrease in genetic diversity may lead to the loss of the ability of adaptation to environmental changes and evolutionary capabilities (Lande, 1993; Kaljund and Jaaska, 2010). Therefore, the protection and maintenance of genetic diversity has important theoretical and practical implications for the protection and utilization of plant resources and genetic improvement of forest tree species. The genetic diversity and structure of populations or species can be attributed to multiple factors, such as distribution ranges, life forms, breeding systems, seed dispersal mechanisms, evolutionary history, climate factors, and human interference (overexploitation, overgrazing, landslides, and fragmentation) (Hamrick and Godt, 1996; Sarin et al., 2015; Gaisberger et al., 2020; Zhou et al., 2020). Identifying climatic factors affecting genetic diversity may provide insights into genetic conservation of species in the context of rapid global climate change. Many studies have explored the impact of climate change on species distribution and genetic structure, confirming that climate change may indirectly affect species distribution to reduce the richness of genetic resources (Abeysinghe et al., 2000; Tan et al., 2018; Garot et al., 2019; Helmstetter et al., 2020). However, different climatic factors play different roles in this process. So far, little is understood about how precipitation and altitude shape the genetic diversity of species. It is known that *P. emblica* is only distributed in Southern and Southwestern areas in China, both in typical dry climate areas and wet climate areas (Pathak, 2003). In addition, in the field investigation, we observed that this species had larger population sizes and better fruit traits (such as fruit size, color and taste) in dry climate areas than that in wet climate areas. Therefore, we hypothesized that altitude and geographical location as well as the environmental stress caused by the long-term extreme precipitation synthetically made the *P. emblica* populations in dry climate areas have high genetic diversity and environmental adaptability.

SSR markers have been widely used in genetic diversity studies because of their co-dominance, high polymorphism rate, good reproducibility, and rapid excavation at a low cost (Bouck and Vision, 2007; Nasim et al., 2020). Based on 20 highly polymorphic EST-SSR markers developed in our previous study (Liu et al., 2018), we evaluated the genetic structure, bottleneck effect, and effective population size of 10 *P. emblica* populations distributed in typical dry-hot climate areas and typical humid climate areas. We aimed to answer the following questions: (1) What is the level of genetic diversity of *P. emblica* in both of these typical climate areas? (2) What is the relative importance of geographic and environmental distance as well as precipitation and altitude to the genetic diversity of *P. emblica*? (3) What is the population dynamics of the species? We hoped to provide a reference for the protection and rational utilization of the wild resources of *P. emblica* as well as theoretical guidance for the ecological restoration and economic development of mountainous areas in China.

## Materials and Methods

### Plant Materials and Study Sites

**FIGURE 1:**
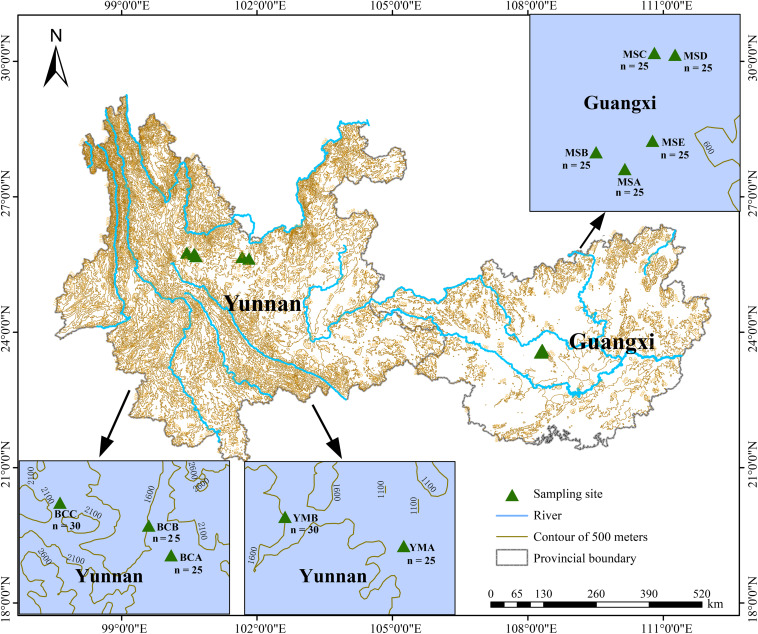
Geographical distribution of the 10 populations of *P. emblica* in this study. The populations YMA, YMB, respectively, represent sampling sites A (25°37′56″N, 101°49′12″E, 1174.8 m a.s.l.) and B (25°40′09″N, 101°40′09″E, 1245.8 m a.s.l.) in Yuanmou County, Yunnan Province; the populations BCA, BCB, and BCC respectively represent sampling sites A (25°41′20″N, 100°38′39″E, 1667.7 m a.s.l.), B (25°44′15″N, 100°36′22″E, 1627.8 m a.s.l.), and C (25°45′57″N, 100°26′28″E, 1742.1 m a.s.l.) in Binchuan County, Yunnan Province; the populations MSA, MSB, MSC, MSD, and MSE respectively represent sampling sites A (23°33′02″N, 108°17′43″E, 253.1 m a.s.l.), B (23°33′31″N, 108°16′53″E, 221.0 m a.s.l.), C (23°36′23″N, 108°18′34″E, 214.2 m a.s.l.), D (23°36′20″N, 108°19′10″E, 339.2 m a.s.l.), and E (23°33′51″N, 108°18′31″E, 242.5 m a.s.l.) in Mashan County, Guangxi Province. The number of samples is detailed in the map of each population.

**FIGURE 2:**
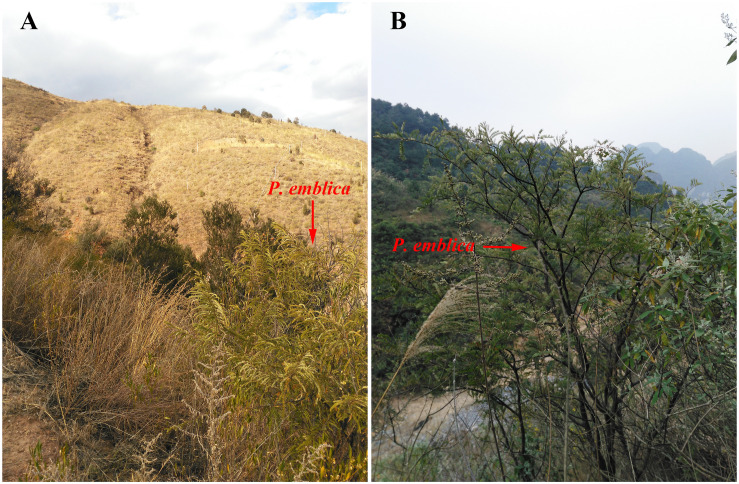
The habitats of *P. emblica* distributed in dry-hot valley of Yunnan **(A)** and humid areas of Guangxi **(B)**.

A total of 260 *P. emblica* samples were collected from 10 wild populations (YMA, YMB, BCA, BCB, BCC, MSA, MSB, MSC, MSD, and MSE) which were distributed in two main climate areas: arid areas (Yuanmou County and Binchuan County in Yunnan) and humid areas (Mashan County in Guangxi) (Figures 1, 2). The altitude and 12 bioclimatic factors (Fick and Hijmans, 2017) showed significant differences between these two climate areas (Supplementary Table S1). The five populations in the Yunnan dry-hot area were located above 1000 m, whereas the five populations in the humid area of Guangxi were all located below 500 m. The average annual precipitation in the area of the five populations in Yunnan was lower than 900 mm, whereas that in the area of the five populations in Guangxi was higher than 1700 mm. In addition, in order to avoid repeated clone sampling, the sampled individuals in every population were at least 15 m apart from each other when collecting leaves. The collected leaf tissues were quickly dried with silica gel and stored at −20°C until further analyses.

### DNA Extraction and EST-SSR Genotyping

Genomic DNA was isolated with the modified CTAB method (Doyle and Doyle, 1987). All samples were genotyped using 20 EST-SSR markers previously developed for the species (Liu et al., 2018). For all markers, PCR amplification (three replicates of each sample) was performed on ABI 2720 Thermal Cycler (Thermo Fisher Scientific, Waltham, MA, United States). The 15 μL reaction mixture contained 1 μL (15–20 ng) of genomic DNA, 7.5 μL of 2 × PCR Master Mix (TSINGKE, Beijing, China), 1 μL (10 pM) of each primer, and 4.5 μL of ddH2O. The PCR program was as follows: 5 min at 94°C; 30 cycles of 30 s at 94°C, annealing at 58°C for 30 s, 30 s at 72°C; and a final extension step at 72°C for 5 min. The PCR products were pooled and purified using the Wizard SV Gel and PCR Clean-Up System (Promega) following the manufacturer’s instructions. The 5′ end of each forward primer for the 20 markers was tagged with one of three fluorescent dyes (FAM, HEX, or ROX), and multiplex PCR amplifications were performed for the 260 individuals of *P. emblica* using the PCR conditions described above. Allele sizes for the tagged PCR products were obtained using an ABI 3730 sequencer with a GeneScan 500 LIZ Size Standard and GeneMapper 4.1 (Thermo Fisher Scientific, Waltham, MA, United States).

### Data Analysis

POPGENE version 1.31 (Yeh et al., 1999) was used to calculate the average number of alleles (*N*a), the effective number of alleles (*N*e), Shannon’s diversity index (*I*), the observed heterozygosity (*H*o), the expected heterozygosity (*H*e), inbreeding coefficient (*F*_*IS*_), outcrossing rate *t* [*t* = (1 − *F*_*IS*_)/(1 + *F*_*IS*_)] (Kesseli, 1992), gene flow (*N*m), percentage of polymorphic loci (*PPL*), and genetic differentiation index (*F*_*ST*_). Polymorphic information (PIC) was calculated using PowerMarker version 3.25 (Liu and Muse, 2005).

The standard WorldClim bioclimatic variables with a spatial resolution of 30 s (∼1 km^2^) were obtained from WorldClim version 2 (Fick and Hijmans, 2017), and they were the average values for the period of 1970–2000. Based on the latitude and longitude information obtained in the field, the bioclimatic variables of the 10 investigated populations were extracted using ArcGIS version 10 (ESRI, 2012). Principal component analysis (PCA) of altitude and bioclimatic variables of the 10 populations was conducted using the FactoMineR package (Sébastien et al., 2008).

Mantel and partial Mantel tests were performed using the R vegan package to assess the relationships between genetic diversity indices (Bray–Curtis distance) and geographical distance or environmental variables (Euclidean distance) with 9999 permutations. To further explore the relative importance of altitude and precipitation to genetic diversity, we also conducted a similar correlation analysis with them as environmental distance (Euclidean distance), respectively.

In order to evaluate the population genetic structure of *P*. *emblica*, several approaches were used in this study. Firstly, the Bayesian clustering analysis of the population structure was carried out using STRUCTURE version 2.3.4 (Pritchard et al., 2000). The program was run with the admixture model and independent-allele frequency using a burn-in period of 3 × 10^5^ and 1 × 10^5^ Markov chain Monte Carlo (MCMC) replications. A total of 20 independent runs were performed for each *K* value ranging from 1 to 20. The most likely number of clusters was evaluated using both Δ*K* and the log-likelihood value with STRUCTURE HARVESTER version 0.6.94 (Evanno et al., 2005; Earl and VonHoldt, 2012). Furthermore, the hierarchical analysis on 260 *P. emblica* individuals was performed in the R stats package with UPGMA algorithm based on the Hellinger distance. Following this, cluster analysis on the basis of shared allele distance (DAS) was performed using POPULATIONS version 1.2.30 (Langella, 2007) with the neighbor-joining (NJ) method. The NJ tree was visualized and edited in iTOL version 3 (Letunic and Bork, 2016). Finally, based on Nei’s genetic distance (Nei, 1973), principal coordinate analysis (PCoA) of 260 individuals was conducted with ape package version 5.4 (Paradis and Schliep, 2019).

Analysis of molecular variance (AMOVA) was carried out to estimate the partitioning of genetic variation between and within the populations using ARLEQUIN version 3.0 (Excoffier et al., 2005). The effective population sizes for the 10 populations were calculated using LDNE version 1.31 (Waples and Do, 2008) at three levels of lowest allele frequency (*P_*crit*_* = 0.05, 0.02, 0.01) at 95% confidence interval (CI). In order to gain insight into the population dynamic history of *P. emblica*, the ABC framework in DIYABC v. 2.0 (Cornuet et al., 2014) was used to test the applicability of seven plausible scenarios based on 20 highly polymorphic SSR loci, and the posterior probability of the optimal model was estimated with two methods: direct estimate and logistic regression. The scenario with the highest posterior probability was selected, and the prior settings of the detailed parameters used to simulate the DIYABC scenarios are shown in Supplementary Table S2. Times and effective population sizes are not strictly to scale. Since DIYABC analysis adopts generation numbers, the generation times of *P. emblica* was assumed to be 5 years and were converted to a calendar year.

## Results

### Genetic Diversity

A total of 562 alleles were detected in the 20 analyzed SSR loci. The number of alleles (*N*a), expected heterozygosity (*H*e), and Shannon’s diversity index (*I*) ranged from 18 to 40, 0.485 to 0.921, and 1.324 to 2.851, respectively, with the means of 28.1, 0.796, and 2.232, respectively (Table 1), indicating that these 20 SSR makers were highly polymorphic. Polymorphic information content (PIC; Table 1), which was also used to evaluate loci polymorphisms (Xie et al., 2010; Wang et al., 2014), showed that 19 loci were highly polymorphic (PIC > 0.5), and only one locus (PE14171) was moderately polymorphic (0.25 < PIC < 0.5), further indicating high polymorphism of these 20 loci.

**TABLE 1:** Genetic diversity parameters of the 20 EST-SSR makers in all tested individuals of *P. emblica.*

Locus	*N*a	*N*e	*H*o	*H*e	*I*	*F*_*IS*_	*F*_*ST*_	PIC
PE399	25	8.230	0.702	0.881	2.505	0.141	0.062	0.868
PE788	30	9.789	0.849	0.900	2.703	0.004	0.045	0.891
PE4618	29	5.125	0.850	0.806	2.171	−0.104	0.032	0.784
PE6781	29	6.134	0.784	0.839	2.305	0.011	0.041	0.822
PE6950	18	3.267	0.572	0.695	1.601	0.144	0.030	0.653
PE7362	25	3.412	0.405	0.708	1.909	0.343**	0.112	0.691
PE7779	33	6.690	0.638	0.852	2.353	0.146*	0.113	0.837
PE8467	23	7.899	0.756	0.875	2.386	0.053	0.080	0.861
PE8480	32	11.694	0.573	0.916	2.765	0.317***	0.086	0.908
PE9600	29	4.440	0.508	0.776	2.153	0.238**	0.153	0.759
PE10156	23	4.230	0.883	0.765	1.940	−0.211	0.036	0.742
PE10572	31	4.038	0.781	0.754	1.948	−0.092	0.048	0.720
PE11297	28	4.563	0.715	0.782	2.061	0.014	0.070	0.755
PE14171	19	1.938	0.204	0.485	1.324	0.502**	0.152	0.476
PE14389	32	2.233	0.298	0.553	1.617	0.398**	0.082	0.543
PE14485	24	12.292	0.442	0.921	2.784	0.471***	0.078	0.913
PE15252	27	8.947	0.685	0.890	2.545	0.184*	0.060	0.879
PE17379	37	9.076	0.643	0.892	2.777	0.230**	0.058	0.882
PE17828	40	11.003	0.689	0.911	2.856	0.173**	0.090	0.903
PE21382	28	3.928	0.357	0.747	1.936	0.331***	0.290	0.719

Na, number of alleles; *N*e, effective number of alleles; Ho, observed heterozygosity; *H*e, expected heterozygosity; *I*, Shannon’s diversity index; F_*IS*_, inbreeding coefficient; F_*ST*_, genetic differentiation coefficient; PIC, polymorphism information content. *p < 0.05, significant difference; **p < 0.01, extremely significant difference; ***p < 0.001, the most significant difference.

At the population level, the percentages of polymorphic loci (*PPL*) in all 10 populations were 100%; however, the genetic diversity of each population was different. The YMB population had the highest genetic diversity (*H*e = 0.819), whereas MSE had lowest genetic diversity (*H*e = 0.604) (Table 2). Moreover, the mean genetic diversity of the 10 populations investigated in the present study (*H*e = 0.792) was at a high level; however, the average genetic diversity of the five populations of *P*. *emblica* from dry climate areas of Yunnan (*H*e = 0.786) was higher than that of the five populations from wet climate areas of Guangxi (*H*e = 0.673) (Table 2). In addition, at the species level, we found that the genetic diversity (*H*e = 0.796) of *P*. *emblica* was also at a high level (Table 2).

**TABLE 2:** Genetic diversity in 10 populations of *P. emblica.*

	Pop	*N*a	*N*e	*H*o	*H*e	*I*	*F*_*IS*_^*a*^	*t*	*F*_*ST*_	*N*m	*PPL* (%)
The dry climate group	YMA	13.150	6.192	0.678	0.795	2.040	0.148	0.743	−	−	100
	YMB	10.950	6.020	0.606	0.819	1.994	0.261*	0.587	−	−	100
	BCA	11.650	5.352	0.440	0.754	1.890	0.228**	0.629	−	−	100
	BCB	12.000	5.759	0.578	0.777	1.954	0.256*	0.593	−	−	100
	BCC	11.200	5.422	0.586	0.784	1.931	0.253*	0.596	−	−	100
	Population level	11.790	5.749	0.578	0.786	1.962	0.229	0.629	0.042	5.706	100
The wet climate group	MSA	8.600	3.934	0.660	0.661	1.489	0.002	0.996	−	−	100
	MSB	8.150	3.975	0.679	0.664	1.492	−0.024	1.048	−	−	100
	MSC	9.000	4.274	0.612	0.666	1.547	0.080	0.852	−	−	100
	MSD	11.450	5.977	0.280	0.768	1.911	0.212**	0.650	−	−	100
	MSE	8.100	3.367	0.608	0.604	1.335	−0.006	1.012	−	−	100
	Population level	9.060	4.305	0.568	0.673	1.555	0.053	0.912	0.071	3.255	100
	Mean	10.425	5.027	0.573	0.792	1.758	0.141	0.770	−	−	100
	Species level	28.100	6.446	0.617	0.796	2.232	0.150	0.739	0.084	2.744	100

Na, average number of alleles; *N*e, effective number of alleles; *H*o, observed heterozygosity; He, expected heterozygosity; *I*, Shannon’s diversity index; F_*IS*_, inbreeding coefficient; t, outcrossing rate; *F*_*ST*_, genetic differentiation coefficient; Nm, gene flow; PPL, percentage of polymorphic loci. ^*a*^Significant deviation from Hardy–Weinberg equilibrium after sequential Bonferroni corrections: *p < 0.05, **p < 0.01.

### Effects of Geographical and Environmental Distances on Genetic Diversity Indices of 10 *P. emblica* Populations

**FIGURE 3:**
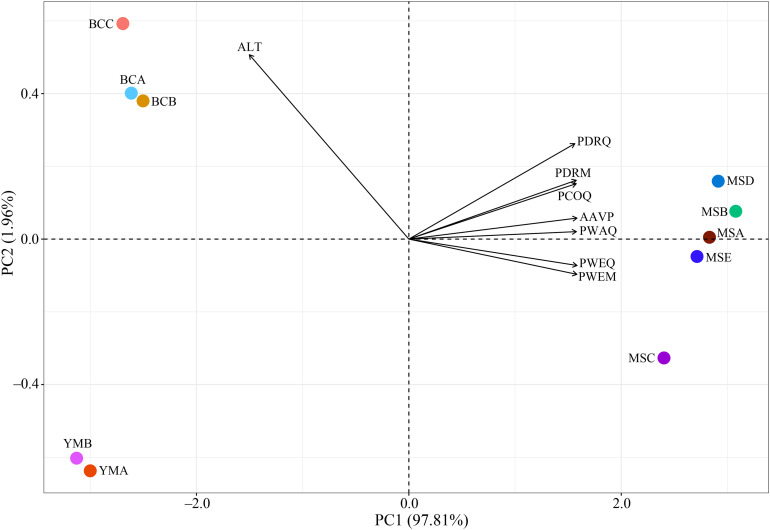
Principal component analysis (PCA) of environmental variables of 10 *P. emblica* populations. AAVP, annual average precipitation (mm); PWEQ, precipitation of the wettest quarter (mm); PDRQ, precipitation of the driest quarter (mm); PWEM, precipitation of the wettest month (mm); PDRM, precipitation of the driest month (mm); PWAQ, precipitation of the warmest quarter (mm); PCOQ, precipitation of the coldest quarter (mm); ALT, altitude (m). All environmental variables were normalized by *z*-score.

The PCA results show that PC1 (97.81%) not only divided the seven precipitation-related bioclimatic variables and altitude into two clusters, but also made a clear distinction between the populations in dry and wet climatic areas (Figure 3). The altitude might be the main factor of environmental difference between these two climatic areas, showing a positive effect in the dry climatic area, whereas the precipitation-related bioclimatic variables showed a positive effect in the wet climatic area.

**FIGURE 4:**
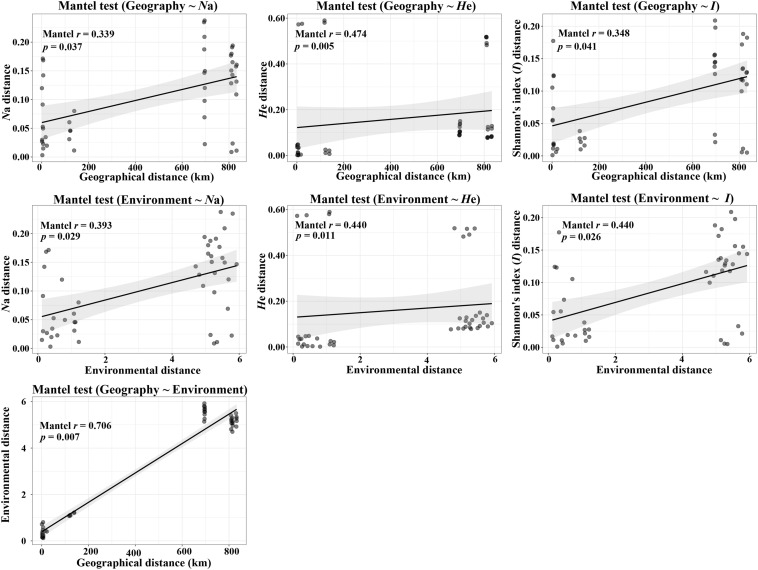
Plot of Mantel tests for the correlation between genetic diversity parameter (Bray–Curtis distance) and explanatory distances (geographic and environmental distance) using Spearman’s coefficient.

**FIGURE 5:**
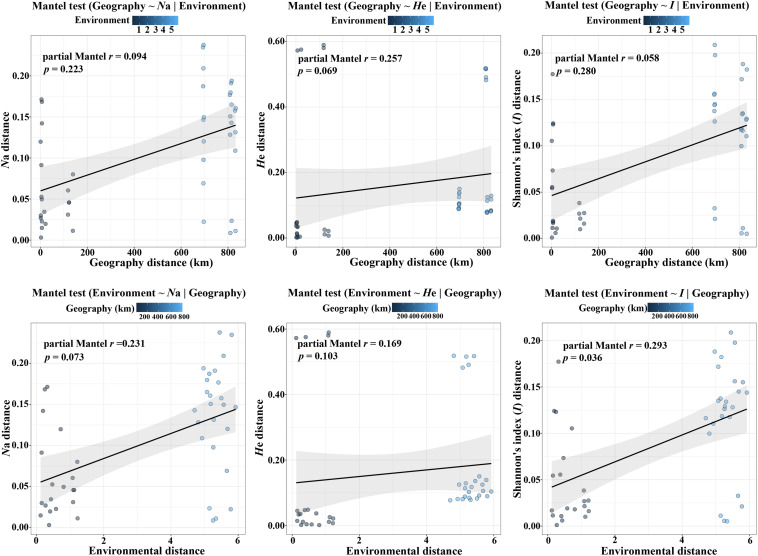
Plot of partial Mantel tests for the correlation between genetic diversity parameter (Bray–Curtis distance) and explanatory distances (geographic and environmental distance) using Spearman’s coefficient.

According to the Mantel tests, both environmental distance and geographic distance had significant effects on three genetic diversity indices (Figure 4). Compared with geographic distance, environmental distance had a stronger effect on average number of alleles (*N*a) and Shannon’s diversity index (*I*), while geographic distance explains more variation of expected heterozygosity (*H*e) (Figure 4). In addition, there was a significant correlation between geographic distance and environmental distance. However, according to the partial Mantel tests, only the pure effect of environmental distance on Shannon’s information index (*I*) was significant (Figure 5; *p* = 0.036 < 0.05).

Furthermore, the Mantel tests of precipitation and altitude showed that altitude had a greater impact on genetic diversity parameters than precipitation (Table 3 and Supplementary Figures S1, S2). In addition, under controlling for geographic distance, altitude was significantly related to both average number of alleles (*N*a) and Shannon’s diversity index (*I*), while precipitation was only significantly related to Shannon’s diversity index (*I*) and its effect was weaker than that of altitude (Table 3 and Supplementary Figure S2). These results implied that both altitude and precipitation had significant effects on the genetic diversity of *P. emblica*, however, compared with precipitation, the pure effect of altitude was slightly stronger.

**TABLE 3:** Mantel and partial Mantel tests for the correlation between genetic diversity parameter (Bray–Curtis distance) and explanatory distances (geographic and environmental (altitude or precipitation) distance) using Spearman’s coefficient.

Effects of	Controlling for	*N*a	*H*e	*I*	Geo
Alt		0.472*	0.472**	0.442*	0.939***
Alt	Geo	0.475**	0.089	0.356*	
Geo	Alt	−0.344	0.102	−0.216	
Pre		0.395*	0.440**	0.440*	0.674**
Pre	Geo	0.240	0.185	0.297*	
Geo	Pre	0.107	0.268	0.077	

Alt, altitude as environmental distance measured as Euclidean distance; Geo, geographic distance; Pre, precipitation as environmental distance measured as Euclidean distance using seven precipitation-related bioclimatic variables shown in Figure 3; *N*a, average number of alleles; *H*e, expected heterozygosity; *I*, Shannon’s information index. *p < 0.05, **p < 0.01, ***p < 0.001. The significances are tested based on 9999 permutations.

### Genetic Differentiation and Genetic Structure of Populations

The results showed that the genetic differentiation between populations (*F*_*ST*_) was 0.084 (Table 2), indicating a moderate genetic differentiation among the 10 populations (0.05 < *F*_*ST*_ < 0.15). The populations YMB, BCA, BCB, BCC, and MSD had significant deviations from the Hardy-Weinberg equilibrium (*p* < 0.05 or *p* < 0.01) (Table 2). The inbreeding coefficients (*F*_*IS*_) of these five populations were positive; correspondingly, their outcrossing rates (*t*) were < 1 (Table 2), indicating the presence of inbreeding in these five populations.

**FIGURE 6:**
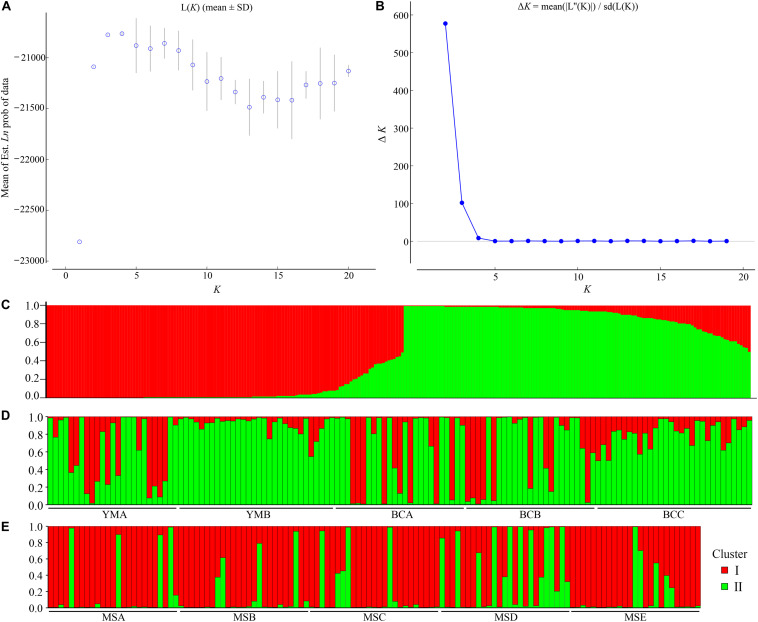
Bayesian model-based clustering STRUCTURE analysis of 260 individuals of *P. emblica*. **(A)** Mean log-likelihood [Ln(*K*) ± SD] against the number of *K*; **(B)** Δ*K* values for different numbers of clusters (*K*); **(C–E)** Estimated population structure of 260 *Phyllanthus emblica* individuals on *K* = 2: **(C)** bar plot was sorted by *Q*-values in single line, and **(D,E)** bar plot was grouped by population ID in multiple lines.

**FIGURE 7:**
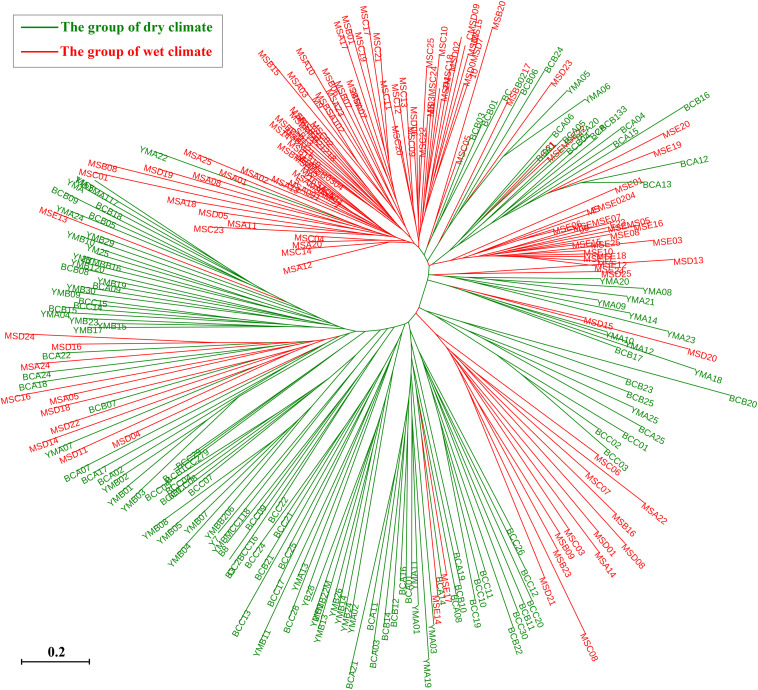
Neighbor-joining tree based on shared allele distance (DAS) between 260 individuals of *P. emblica*.

**FIGURE 8:**
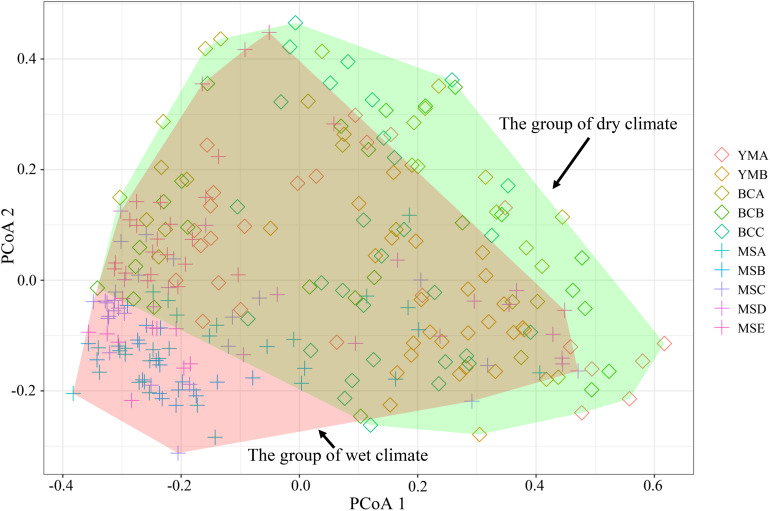
Principal coordinate analysis (PCoA) of 260 *P. emblica* individuals from 10 populations, based on a genetic distance matrix of 20 EST-SSRs. The PCoA1 explains 6.54% of the total genetic variance, and the PCoA2 accounts for 2.91%. The confidence interval of 95% is showed as polygon.

The STRUCTURE analysis showed that Δ*K* reached the maximum value (Δ*K* = 577.307) at *K* = 2 (Figures 6A,B), which suggested that the 260 individuals from the 10 populations most likely belonged into two principal genetic clusters (Figure 6C). Cluster II mainly included individuals from the dry climate group, containing YMA, YMB, BCA, BCB, and BCC populations, whereas cluster I consisted of most of the individuals from the wet climate group, containing MSA, MSB, MSC, MSD, and MSE populations (Figures 6D,E). However, there were some individuals with admixed genotype in both two groups, indicating ongoing gene flow or weak genetic differentiation. Although L(*K*) has the highest value and least variance at *K* = 4 (Figure 6A), the division of genetic clusters was unclear and the gene introgression between populations was relatively disordered (Supplementary Figure S3), which could not reasonably explain the background of the sampling populations. The result of the hierarchical analysis showed that 260 individuals were divided into two main groups (dry and wet climate groups) at the distance of 0.02 (Supplementary Figure S4). The neighbor-joining tree showed that 260 individuals were grouped into two principal clades (Figure 7). One clade included the majority of the individuals from the dry climate group, and the other clade mainly included the individuals from the wet climate group. However, the PCoA results show that PCoA1 and PCoA2 explained 6.54% and 2.91% of the total genetic variance, respectively (Figure 8).

At the species level, the AMOVA revealed that the majority of the observed genetic variation could be attributed to differences within populations (92.44%) rather than to the variation among populations (7.56%) (Table 4). At the group level, higher genetic variation was observed within populations than among populations in both climate groups; however, the genetic variation within the populations from the dry climate group (96.24%) was higher than that within the populations from the wet climate group (93.36%) (Table 4). There was moderate genetic differentiation between the two climate groups (*F* = 0.095); 4.66% of the genetic variations were attributed to the differences among the groups, 90.53% were attributed to the differences within populations, and 4.81% were attributed to the differences among populations within groups (Table 4).

**TABLE 4:** Analysis of molecular variance (AMOVA) based on 20 EST-SSR markers for populations of *P. emblica*.

	Source of variation	d.f.	Sum of squares	Variance components	Percentage of variation	*F*
Whole	Among populations	9	357.352	0.619 Va	7.560	0.076**
	Within populations	510	3857.863	7.564 Vb	92.440	−
	Total	519	4215.215	8.183	−	−
The dry climate group:	Among populations	4	100.955	0.318 Va	3.760	0.038**
YMA, YMB, BCA, BCB, BCC	Within populations	265	2153.963	8.128 Vb	96.240	−
	Total	269	2254.919	8.446	−	−
The wet climate group:	Among populations	4	126.808	0.495 Va	6.640	0.066**
MSA, MSB, MSC, MSD, MSE	Within populations	245	1703.900	6.955 Vb	93.360	−
	Total	249	1830.708	7.450	−	−
Two groups	Among groups	1	129.589	0.389 Va	4.660	0.095**
	Among populations within groups	8	227.763	0.402 Vb	4.810	0.050**
	Within populations	510	3857.863	7.564 Vc	90.530	0.047**

F, fixation Index. **p < 0.01, extremely significant difference.

### Effective Population Size and Population Dynamic History Based on ABC Model

**FIGURE 9:**
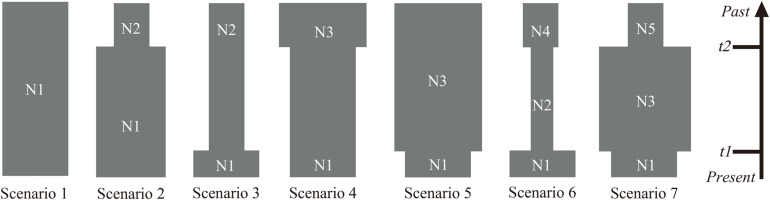
Schematic representation of the seven demographic scenarios (including model parameters) for *P. emblica* tested by the DIYABC method using the SSR loci. (Scenario 1) constant effective population size (N1); (Scenario 2) an old expansion (N2 to N1, t2); (Scenario 3) a recent expansion (N2 to N1, t1); (Scenario 4) an old bottleneck (N3 to N1, t2); (Scenario 5) an recent bottleneck (N3 to N1, t1); (Scenario 6) an old bottleneck (N4 to N2, t2) followed by a recent expansion that led to current effective size (N2 to N1, t1); and (Scenario 7) an old expansion (N5 to N3, t2) followed by a recent bottleneck (N3 to N1, t1).

**FIGURE 10:**
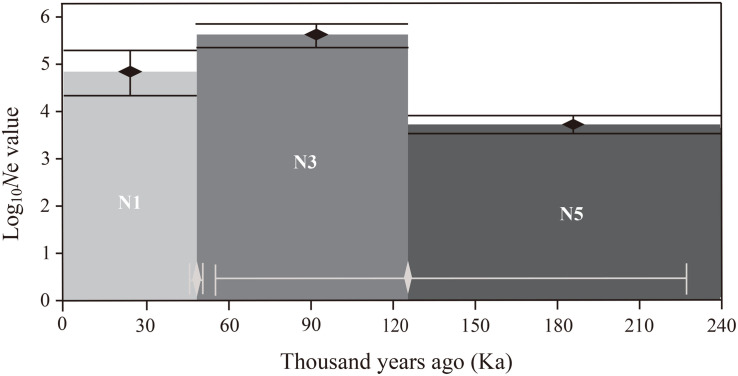
Schematic representation for the estimates of effective population size and the effective population size transition time of the best DIYABC scenario (Scenario 7). Black vertical bars and white horizontal bars represent 95% confidence intervals for the estimates of effective population size and effective population size transition time, respectively. Note that the *y*-axis of effective population size value is in log_10_ format.

Estimations of effective population sizes with the lowest allele frequency (*P*_*crit*_) are listed in Table 5. At *P*_*crit*_ = 0.02 and *P*_*crit*_ = 0.01, effective population size of BCC was the largest, whereas MSA had the smallest effective population size. The DIYABC results showed that the optimal model was Scenario 7 (Supplementary Figure S5), i.e., an ancestral range expansion (the ancestral range expansion was the ancient expansion in Figure 9) followed by a recent range bottleneck (Figure 9). The posterior probabilities obtained from this scenario under direct estimate and logistic regression were 76.25% and 93.60%, respectively; followed by Scenario 6, with posterior probabilities (15.80% and 6.00%). In addition, Scenario 7 had the lowest average type I and type II error rates (14% and 0%, respectively) (Supplementary Table S3). The posterior distribution of the dynamic historical parameters evaluated under the best model (Scenario 7) showed that the initial effective population size of the studied *P. emblica* populations was 8980 (95% confidence interval 7120–9950) individuals (N5; Figure 10 and Supplementary Table S4), and there was a strong expansion at 126 (55–227.50) Ka, resulting in the effective population size up to 53.79 (25.28–76.50) times (N3) of the original. Next, a certain degree of bottleneck appeared at 49.85 (49.55–50.00) Ka, and the effective population size was further reduced to 0.2 (0.04–0.39) times (N1; Figure 10 and Supplementary Table S4) that of the expansion period. Although the 95% confidence interval was wide, the initial expansion seems to occur in the Last Interglacial (LIG), whereas the most recent bottleneck occurred in the beginning of the Last Glacial Maximum (LGM).

**TABLE 5:** The effective population size with three levels of the lowest allele frequency (*P*_*crit*_) for the 10 populations of *P. emblica*.

Population	*P_*crit*_* = 0.05 (95% CIs)	*P_*crit*_* = 0.02 (95% CIs)	*P_*crit*_* = 0.01 (95% CIs)
YMA	8.0 (7.0–9.1)	44.9 (39.4–52)	44.9 (39.4–52)
YMB	29.0 (24.1–35.8)	54.4 (43.5–71.2)	54.4 (3.5–71.2)
BCA	10.6 (9.2–12.4)	39.1 (34.0–45.7)	39.1 (34.0–45.7)
BCB	15.1 (13.0–17.7)	67.0 (54.9–85.0)	67.0 (54.9–85.0)
BCC	24.4 (20.2–30.2)	83.5 (60.4–90.0)	83.5 (60.4–90.0)
MSA	9.7 (7.9–11.8)	10.1 (9.2–11.2)	10.1 (9.2–11.2)
MSB	15.2 (12.4–19)	22.1 (19.2–25.6)	22.1 (19.2–25.6)
MSC	9.3 (7.7–11.1)	12.2 (11.1–13.5)	12.2 (11.1–13.5)
MSD	9.4 (8.3–10.7)	25.0 (22.6–27.8)	25.0 (22.6–27.8)
MSE	10.9 (8.7–13.7)	11.7 (10.5–13.0)	11.7 (10.5–13.0)

## Discussion

### High Genetic Diversity Within Populations and Low Genetic Differentiation Among Populations

The evolutionary potential of a species and its ability to withstand adverse environments depend on both genetic diversity of the species and its population genetic structure (Stebbins, 1950). Different levels of genetic variation and genetic structure of populations can be attributed to multiple factors, such as distribution ranges, life forms, breeding systems, seed dispersal mechanisms, evolutionary history, natural selection, and human interference (Hamrick and Godt, 1996; Sarin et al., 2015; Zhou et al., 2020). In general, the genetic diversity of widespread plant species is higher than that of narrowly distributed species (Hamrick and Godt, 1996), and compared to annual and perennial herbaceous plants, perennial woody plants tend to have higher genetic diversity at the species level and within populations, but lower genetic variation among populations (Hamrick et al., 1979, 1992; Chung et al., 2020). As a long-lived perennial woody plant widely distributed in tropical and subtropical areas, *P. emblica* was found to have high genetic diversity at the species level (*H*e = 0.796), and the mean genetic diversity of the 10 populations investigated in the present study (*H*e = 0.792) was similar to that reported for this species in Thailand (*H*e = 0.619) (Pandey and Changtragoon, 2012) and Sri Lanka (*H*e = 0.753) (Mawalagedera et al., 2014), but significantly higher than that reported by Nybom (2004) for multiple plant species (*H*e = 0.22 or 0.23). Compared to congeneric herbs, such as *P. amarus* (total diversity *h*_*T*_ = 0.62; average intrapopulation diversity *h*_*S*_ = 0.47; level of genetic differentiation *G*_*ST*_ = 0.24) and *P. fraternus* (*h*_*T*_ = 0.77; *h*_*S*_ = 0.57; *G*_*ST*_ = 0.26) (Sarin et al., 2015), *P. emblica* showed higher genetic variation at the species level (0.796) and within populations (0.792), but lower genetic differentiation among populations (*F*_*ST*_ = 0.084) in the present study. Outcrossing species usually have higher genetic diversity than inbreeding species (Hamrick and Godt, 1989; Nybom, 2004). A previous study revealed that the mating system of *P. emblica* is dominated by outcrossing (Singh et al., 1998). In the present study, the MSB and MSE populations were mainly outcrossing populations (*F*_*IS*_ < 0, *t* > 1); however, for the other eight populations, the inbreeding coefficients (*F*_*IS*_) were positive, and the outcrossing rates (*t*) were lower than 1, indicating insufficient heterozygotes and the possibility of inbreeding. According to our long-term field investigations, this could be attributed to human interference in the habitats of these eight populations, resulting in insufficient heterozygotes and a high inbreeding coefficient. This might also be attributed to hybridization (inbreeding) between adjacent individuals with the same genotype. The richness of plant genetic diversity is related to the complexity of its habitat and ecological factors such as precipitation, temperature, and soil (Abeysinghe et al., 2000), which is a possible reason for different genetic diversity of the same species in different habitats. The high genetic diversity of species with a high inbreeding coefficient may be related to the heterogeneity and complexity of their habitat. In China, *P. emblica* is a widely distributed species and occupies different habitats, which could create a rich gene pool through long-term adaptive evolution, leading to increased genetic diversity. In addition, it is also possible that these populations exhibit a high degree of genetic diversity from ancestral populations originating from southeastern tropical Asia (Morton, 1987; Setoguchi et al., 2011).

Gene flow plays an important role in population genetic differentiation. Genetic migration causes an increase in genetic variation within populations and a decrease in differentiation among populations, which is the opposite of the genetic drift effect (Whitlock and McCauley, 1999). In addition, the genetic differentiation of natural populations is thought to be a dynamic process which relies on the equilibrium relationship between gene flow and selection (Whitlock and McCauley, 1999). In the present study, the populations YMA, MSA, MSB, MSC, and MSD had not significant deviations from the Hardy-Weinberg equilibrium, the estimation of gene flow (*N*m) of these populations of *P. emblica* was >1, indicating that gene flow was high enough to resist the genetic differentiation among populations caused by genetic drift, thus achieving a homogenizing effect (Wright, 1949).

STRUCTURE analysis, NJ tree, and hierarchical analysis revealed that the 260 investigated individuals were grouped into two genetic clusters, and this genetic structure was consistent with geographical distribution, except for some admixed genotypes, which may be a consequence of high genetic variation within populations, resulting in the occurrence of similar genetic variations in the individuals of the two groups. This may be also attributed to genetic migration between the two groups, or to some genes from the ancestral populations shared in the two groups. Pollen spread and seed dispersal are the most important determinants of gene flow. Woody species, especially trees, usually have low population density and large statures, which is beneficial to the long-distance migration of pollen and seeds (Paffetti et al., 1996). In nature, *P. emblica* is mainly pollinated by wind and bees (Bajpai, 1957), and the long-distance transmission of pollen provides the chances of long-distance genetic migration between different populations (Nakanishi et al., 2020). In nature, *P. emblica* usually depends on seed propagation, and long-distance seed dispersal must depend on certain fruit-eating animals (Li and Zhao, 2007). Because of the limited range of activities of fruit-eating animals and the limited number of seeds they are able to carry, the contribution of seed dispersal to the homogenization of *P. emblica* populations is expected to be small. We presumed that the frequent gene flow among *P. emblica* populations might be caused by pollen flow by wind or pollinators. Therefore, we concluded that the investigated species exhibited high levels of genetic diversity and low levels of genetic differentiation.

### Differences Between the Genetic Diversity of *P. emblica* Populations From the Two Climate Areas

According to the niche breadth variation hypothesis, molecular genetic variation is related to environmental variation, and high level of genetic variation is an adaptive strategy of plants for responding to heterogeneous environments (Levins, 1968). The current research supported the original hypothesis, i.e., the level of genetic diversity in the dry climate group (*N*a = 11.790, *H*e = 0.786, *I* = 1.962) was higher than that in the wet climate group (*N*a = 9.060, *H*e = 0.673, *I* = 1.555), and both of these values were higher than the genetic diversity of *Camellia nitidissima* at the population level (Li et al., 2020; *N*a = 3.881, *H*e = 0.546, *I* = 0.988). Moreover, the AMOVA suggested that genetic variation within the populations of the dry climate group (96.24%) was also higher than that within the populations of the wet climate group (93.36%). The Mantel tests confirmed that the genetic diversity of this species was affected by altitude, precipitation and geographic distance. Interestingly, the partial Mantel tests showed that the pure effects of altitude and precipitation only on Shannon’s diversity index (*I*) was significant, which implied that this genetic diversity index was more sensitive to the environmental response to some extent. Just as it is widely used in microbial and forest landscape researches (Allen et al., 2009; Trivedi et al., 2020), Shannon’s diversity index (*I*) is also a key indicator for genetic diversity assessment of woody plants. This study supported the conclusion that altitude significantly affects the distribution of genetic variation in populations (Chen et al., 2008; Hahn et al., 2012). In this study, the populations in low-altitude wet climate areas had lower genetic diversity. On the one hand, it might be because of undergoing stronger genetic bottlenecks and founder effects for these populations (possible range contractions and expansions during the Pleistocene as supported by Scenario 7) (Hensen et al., 2012). On the other hand, it might also be explained by the restriction of sexual reproduction, as the yield and quality of *P. emblica* populations in the wet climate areas were significantly lower than those in the wet climate areas (Liu et al., unpublished data). Recent studies have proposed that genetic diversity basically exhibited a high-low-high pattern with increasing altitude (Wang et al., 2019). However, this study did not involve the genetic diversity analysis of the populations distributed at intermediate altitudes. However, the characteristics of higher genetic diversity in the populations at high altitudes lay a solid foundation for carrying out artificial assisted transplantation of this species in the context of climate warming. As far as precipitation is concerned, according to the statistical data of 79 vascular plants, the precipitation and seasonal drought had important implications for the genetic diversity of vascular plants species (Tan et al., 2018). Although drought is a limiting factor for plant growth, after long-term adaptive evolution, plants in arid environments can developed different drought-resistant mechanisms (such as resistance genes) as well as genetic structures for adaptation to the environment (Ceccarelli and Grando, 1996; Ricardo-Trejo and O’Connell, 2005). The genetic variation and diversity of the populations in arid environments is predicted to increase as a consequence of drought stress, and the rate of evolution of plant populations in these environments is higher than that of the populations in humid environments (Stebbins, 1952). Therefore, this can explain why *P. emblica* in the dry-hot valley of Yunnan had higher genetic diversity than that in the humid area of Guangxi. In addition, it is worth noting that the Mantel tests also revealed the significant correlation between environmental and geographical distance, implying a certain degree of isolation by distance (IBD). Given that intermediate populations between the two climate zones were not sampled and compared, in future researches, further work should be carried out to study the populations distributed in more climatic gradients, altitude gradients and distance gradients to reveal the general effects of altitude, precipitation and geographical distance on genetic diversity.

Our results showed moderate genetic differentiation between the two climate groups (*F* = 0.095). The genetic differentiation among the populations in the wet climate group (*F*_*ST*_ = 0.071) was higher than that among the populations in the dry climate group (*F*_*ST*_ = 0.042), and it might be the result of combined effects of isolation by distance (IBD) and environmental selection. The effective population size of the dry climate group was larger than that of the wet climate group, and the effective population sizes of BCC was the largest (83.5), whereas MSA had the smallest effective population size (10.1). However, DIYABC analysis suggested the effective population size of *P. emblica* might have contracted in the beginning of the Last Glacial Maximum. The above results supported that genetic differentiation was associated with decline in the effective population size (Frankel et al., 1995).

### Population Dynamics

Compared with the expansion period, the most likely Scenario 7 revealed that the effective population size of *P. emblica* shrank by approximately 0.8 times at 49.85 Ka. Although there was a certain degree of uncertainty in the estimation of population dynamics history in terms of the 95% confidence interval, the detected bottleneck events corresponded well to the beginning of LGM (Clark et al., 2009). The effective population size of *P. emblica* after experiencing a genetic bottleneck was lower than the estimated value of the ancestral population of the model plant Poplar (*Populus*) (Ingvarsson, 2008), implying the intensity of the bottleneck experienced to some extent. After experiencing the bottleneck effect to some extent, the populations of *P. emblica* still maintained high genetic diversity, which may have been a consequence of gene mutation or high gene flow, and consequently, the level of genetic variation remained close to or returned to its original level. However, if human disturbance continues in the future, it will probably result in population decline, habitat fragmentation, or even population isolation of this species, and *P. emblica* may experience the bottleneck effect once again. According to our long-term field investigations, significant deviations from Hardy-Weinberg equilibrium in the YMB, BCA, BCB, BCC, and MSD populations may be a consequence of human activities which negatively influenced the habitats of these five populations, resulting in heterozygote deficiency and high inbreeding coefficient in these populations (Bourguiba et al., 2020). It is worth noting that *P. emblica* is not distributed in Northern China, whereas almost continuous distribution areas are formed in Southern and Southwestern China (Pathak, 2003). Based on dynamic history of this species preliminarily speculated in this study, the unique distribution bottleneck in China may be explained by the detected genetic bottleneck event that occurred in the beginning of LGM.

### Implications for Ecological Restoration

China has abundant resources of *P. emblica*. However, this famous plant species which has important medicinal, commercial, and ecological values is being consumed at a very high and alarming rate, with the increase in human population leading to the increase in the demand for such plant materials. In Yunnan and Guangxi, the natural *P. emblica* forests are commonly being cut down for fodder, fuel, and wood. Because of the negative influence of climate change and human activities, the native vegetation types in the dry-hot *P. emblica* habitat have declined and are almost non-existent. The existing habitat types mainly include semi-savannah vegetation similar to the valley savannah vegetation in Africa (Jin and Ou, 2000), and in some places, they have even become bare lands and barren hills, leading to the destruction of biodiversity and ecosystem stability as well as serious soil erosion. The habitat of *P. emblica* in the humid climate area of Guangxi is karst landform, where plants live in harsh and extremely fragile conditions with serious soil erosion and high ecological sensitivity. Over-exploitation is a serious threat to the survival of this species, and currently, it is difficult to find well-preserved wild *P. emblica* forests in China. At the same time, the harsh and fragile habitats of *P. emblica* are also facing various threats, including climate change and human activities, and it is difficult for the ecosystem to recover naturally after these disturbances. Therefore, it is crucial to protect and rationally utilize the wild resources of *P. emblica* as well as to put efforts into the ecological restoration of its habitats.

*P. emblica* is a species with excellent tolerance to drought and barren soil because of its well-developed roots, and is often used as a pioneer tree species for forestation. Meanwhile, its high genetic diversity provides more possibilities to breed high-quality varieties of this species to be used as an important medicinal economic plant. The genetic resource conservation of local *P. emblica* populations should be considered mainly for the ecological restoration of dry-hot valleys and karst areas. In addition to *in situ* conservation of the populations with high genetic diversity, *ex situ* germplasm collection for different purposes, such as breeding and conservation, is also significant for achieving our goals. Compared to the five populations from the Guangxi area, the five populations from the dry-hot valley of Yunnan had higher genetic diversity, particularly the YMA and YMB populations, and attention should be payed to this difference. Furthermore, given that the genetic variation mainly existed within populations, more individuals should be selected and propagated within populations used for ecological restoration in Yunnan dry-hot valley area and Guangxi karst area. In the present study, *P. emblica* samples were divided into two gene clusters, which should be taken into account when collecting germplasm resources and carrying out ecological restoration. Finally, environmental protection should be advocated in order to increase local farmers’ awareness of the protection of this valuable species, and it should be forbidden to cut down *P. emblica* trees in its ecological restoration areas.

## Conclusion

According to our investigations of the genetic diversity and genetic structure of *P. emblica* in two different typical climate areas of China by polymorphic EST-SSR markers, *P. emblica* had more genetic diversity at the species level and within populations, but less genetic variation among populations. However, the genetic diversity of *P. emblica* in the dry climate area of Yunnan was higher than that in the wet climate area of Guangxi, and both altitude and precipitation had significant pure effects on this result. In addition, we found that *P. emblica* populations had experienced a bottleneck event in the beginning of the Last Glacial Maximum. The findings of this study will provide new ideas and guidance for the protection and rational development and utilization of *P. emblica* resources, and they will also provide a scientific basis for the ecological restoration and economic development in Yunnan dry-hot valley areas and Guangxi karst areas.

## Data Availability Statement

The original contributions presented in the study are included in the article/Supplementary Materials, further inquiries can be directed to the corresponding author/s.

## Author Contributions

HM conceptualized the study. XL, YM, and ZL contributed to the investigation. XL and YW were in charge of data curation. XL and HM wrote the original draft, and HM reviewed. All authors agree to be accountable for the final manuscript.

## Conflict of Interest

The authors declare that the research was conducted in the absence of any commercial or financial relationships that could be construed as a potential conflict of interest.
