# Supplementary material for: Genetic Diversity of Phyllanthus emblica From Two Different Climate Type Areas
Source: Front Plant Sci. 2020 Nov 30;11:580812. doi: 10.3389/fpls.2020.580812 (PMC7734338; doi:10.3389/fpls.2020.580812)
Supplement: Supplementary file 1 [file Data_Sheet_1.docx]

**SUPPLEMENTARY MATERIAL**

**SUPPLEMENTARY FIGURE LEGENDS**

**Supplementary Figure S1 |** Plot of Mantel tests for the correlation between genetic diversity parameter (Bray–Curtis distance) and explanatory distances (geographic and environmental (altitude or precipitation) distance) using Spearman’s coefficient.

**Supplementary Figure S2 |** Plot of partial Mantel tests for the correlation between genetic diversity parameter (Bray–Curtis distance) and explanatory distances (geographic and environmental (altitude or precipitation) distance) using Spearman’s coefficient.

**Supplementary Figure S3 |** Estimated population structure of 260 *P. emblica* individuals on *K* = 4 based on STRUCTURE analysis. (**A**) Bar plot was sorted by Q-values in single line; (**B**) Bar plot was grouped by population ID in multiple lines.

**Supplementary Figure S4 |** Hierarchical analysis of 260 *P. emblica* individuals.

**Supplementary Figure S5 |** The estimation of posterior probabilities under direct estimate and logistic regression.

**SUPPLEMENTARY TABLES**

**Supplementary Table S1 |** Comparison of 19 bioclimatic variables and altitude in the two climate distribution areas of *P. emblica*.

| Variables | Mean ± SD | |  | Variation range | | Significance |
| --- | --- | --- | --- | --- | --- | --- |
|  | The dry climate area | The wet climate area |  | The dry climate area | The wet climate area |  |
| Annual mean temperature (℃) | 18.358 ± 2.934 | 19.601 ± 1.917 |  | 14.258-21.442 | 16.371-21.392 | 0.451 ns |
| Monthly mean diurnal temperature range (℃) | 10.768 ± 0.358 | 7.152 ± 0.538 |  | 10.233-11.133 | 6.733-7.850 | 0.000** |
| Isothermality | 46.895 ± 2.318 | 29.508 ± 1.427 |  | 43.920-49.887 | 28.008-31.292 | 0.000** |
| Standard deviation of temperature seasonal change | 441.880 ± 18.983 | 622.767 ± 17.499 |  | 419.042-460.468 | 592.343-634.502 | 0.000** |
| Max. temperature of the warmest month (℃) | 28.82 ± 2.736 | 30.860 ± 2.269 |  | 25.100-31.700 | 27.100-32.900 | 0.235 ns |
| Min. temperature of the coldest month (℃) | 5.76 ± 2.613 | 6.640 ± 1.491 |  | 1.800-8.300 | 4.100-8.000 | 0.531 ns |
| Range of annual temperature (℃) | 22.960 ± 0.532 | 24.160 ± 0.820 |  | 22.100-23.400 | 23.000-25.000 | 0.025* |
| Mean temperature of the wettest quarter (℃) | 22.736 ± 2.681 | 26.543 ± 2.019 |  | 19.117-25.767 | 23.083-28.217 | 0.035* |
| Mean temperature of the driest quarter (℃) | 13.483 ± 3.592 | 13.030 ± 1.815 |  | 9.233-17.383 | 10.017-14.833 | 0.807 ns |
| Mean temperature of the warmest quarter (℃) | 22.963 ± 2.899 | 26.543 ± 2.019 |  | 19.117-25.950 | 23.083-28.217 | 0.053 ns |
| Mean temperature of the coldest quarter (℃) | 12.410 ± 2.833 | 11.407 ± 1.707 |  | 8.250-15.183 | 8.600-13.167 | 0.517 ns |
| Annual average precipitation (mm) | 743.800 ± 79.462 | 1735.600 ± 28.763 |  | 648.000-814.000 | 1700.000-1767.000 | 0.000 ** |
| Precipitation of the wettest month (mm) | 142.600 ± 2.966 | 295.800 ± 6.723 |  | 138.000-146.000 | 285.000-302.000 | 0.000 ** |
| Precipitation of the driest month (mm) | 8.600 ± 3.782 | 40.000 ± 2.550 |  | 4.000-12.000 | 36.000-43.000 | 0.000 ** |
| CV of precipitation | 80.609 ± 12.955 | 67.803 ± 1.231 |  | 68.526-95.931 | 67.026-69.944 | 0.059 ns |
| Precipitation of the wettest quarter (mm) | 395.000 ± 7.616 | 836.400 ± 11.014 |  | 386.000-405.000 | 824.000-851.000 | 0.000 ** |
| Precipitation of the driest quarter (mm) | 34.800 ± 17.398 | 135.600 ± 9.343 |  | 14.000-51.000 | 120.000-145.000 | 0.000 ** |
| Precipitation of the warmest quarter (mm) | 345.200 ± 52.399 | 836.000 ± 12.062 |  | 303.000-405.000 | 824.000-853.000 | 0.000 ** |
| Precipitation of the coldest quarter (mm) | 35.200 ± 16.814 | 192.600 ± 15.931 |  | 16.000-51.000 | 167.000-206.000 | 0.000 ** |
| Altitude (m) | 1491.640 ± 261.291 | 254.000 ± 50.163 |  | 1174.800-1742.100 | 214.200-339.200 | 0.000 ** |

The dry climate area including sampling sites YMA, YMB, BCA, BCB, BCC, and the wet climate area including sampling sites MSA, MSB, MSC, MSD, MSE. **p* < 0.05, significant difference; ***p* < 0.001, extremely significant difference; ns, no significant difference.

**Supplementary Table S2 |** Prior settings for all parameters used when simulating DIYABC scenarios. Population size parameters are in units of population effective size, while time parameters are in units of generations.

| **Priors for the parameters of** | |
| --- | --- |
| N1 | UN~[5E3 – 2E5] |
| N2 | UN~[10 – 4E3] |
| N3 | UN~[5E3 – 7E5] |
| N4 | UN~[1E2 – 1E4] |
| N5 | UN~[1E2 – 1E4] |
| t1 | UN~[1 - 1E4] |
| t2 | UN~[1E4 – 5E4] |
| Constraint on parameter | N2<N1, t1<t2, N3>N1, N4<N1, N4>N2, N5<N1, N5<N3 |
| *Priors for the mutation model for* *nuclear microsatellites* | |
| MEAN - µ | UN~[1 x 10^-4^ - 1 x 10^-3^] |
| GAM - µ | GA~[1 x 10^-5^, 1 x 10^-2^, 2] |
| MEAN - P | UN~[0.1, 0.3] |
| GAM - P | GA~[1 x 10^-2^ , 9 x 10^-1^, 2] |
| MEAN - SNI | LU~[1 x 10^-8^, 1 x 10^-5^] |
| GAM - SNI | GA~[1 x 10^-9^, 1 x 10^-3^, 2] |
| Max. # steps | 40 |

Uniform distribution (UN) with 2 parameters: min and max; Gamma distribution (GAM) with 4 parameters: min, max, shape; Log-Uniform (LU) distribution with 2 parameters: min and max; Ancestral effective population size (N1); Effective population size before expansion (N2, N5); Effective population size before bottleneck (N3, N4); Ancestral population expansion/ bottleneck time (t2); Recent population expansion/ bottleneck time (t1). Times and population sizes are not to scale.

**Supplementary Table S3 |** Estimates of type I and type II error probabilities for the seven scenarios in DIYABC.

| True scenario used for simulation | 1 | 2 | 3 | 4 | 5 | 6 | 7 |  |
| --- | --- | --- | --- | --- | --- | --- | --- | --- |
|  | Type I Error* | | | | | |  | Type II Error* |
| 1 | – | 0.21 | 0.21 | 0.20 | 0.18 | 0.16 | 0.02 | 0.14 |
| 2 | 0.03 | – | 0.30 | 0.09 | 0.07 | 0.19 | 0.04 | 0.10 |
| 3 | 0.03 | 0.29 | – | 0.10 | 0.11 | 0.14 | 0.05 | 0.10 |
| 4 | 0.04 | 0.14 | 0.11 | – | 0.23 | 0.16 | 0.00 | 0.10 |
| 5 | 0.03 | 0.14 | 0.10 | 0.29 | – | 0.17 | 0.00 | 0.10 |
| 6 | 0.03 | 0.23 | 0.21 | 0.15 | 0.17 | – | 0.02 | 0.12 |
| 7 | 0.00 | 0.01 | 0.00 | 0.00 | 0.00 | 0.00 | – | 0.00 |
| Mean | 0.15 | 1.03 | 0.94 | 0.82 | 0.77 | 0.81 | 0.14 |  |

*Error probabilities were calculated based on simulating 500 datasets with an assumed “true scenario”. Type I errors were estimated as proportions of instances where the chosen scenario did not exhibit the highest posterior probability compared to the competing scenarios. While type II errors were estimated by counting the proportion of data sets that resulted in the highest posterior probability of the chosen scenario, although simulated with other scenarios. Here, the type I error probabilities are listed in the column under each specified scenario. The type II error is obtained by calculating the mean of type I errors across the table for all scenarios. The scenario with the highest posterior probability (76.251%, 93.60%) is shaded in gray.

**Supplementary Table S4 |** Estimates of posterior distributions of parameters revealed from the Approximate Bayesian Computation (ABC) for the best scenarios 7 for the demographic history of *P. emblica*. Estimation is based on 1% of the closest simulated data sets and the logit transformation of parameters was used. Time parameter (t) (unit of time: years) was scaled by an assumed generation time of five years.

| Parameter | Mean | Median | Mode | 5% | 95% |
| --- | --- | --- | --- | --- | --- |
| N1 | 95400 | 89400 | 38500 | 21100 | 186000 |
| N3 | 483000 | 493000 | 687000 | 227000 | 687000 |
| N5 | 8980 | 9220 | 9930 | 7120 | 9950 |
| T1(gen） | 9970 | 9990 | 10000 | 9910 | 10000 |
| T1(Ka) | 49.85 | 49.95 | 50.00 | 49.55 | 50.00 |
| T2(gen） | 25200 | 23200 | 12400 | 11000 | 45500 |
| T2(Ka) | 126.00 | 116.00 | 62.00 | 55.00 | 227.50 |
| Mean umic | 8.99×10-4 | 9.46×10-4 | 9.98×10-4 | 6.39×10-4 | 9.97×10-4 |
| Mean p | 2.91×10-1 | 3.00×10-1 | 3.00×10-1 | 2.96×10-1 | 3.00×10-1 |

Abbreviations: N = effective population size; t = population founding time.
