# Supplementary figures and images for: Genetic Diversity of Phyllanthus emblica From Two Different Climate Type Areas
Source: Front Plant Sci. 2020 Nov 30;11:580812. doi: 10.3389/fpls.2020.580812 (PMC7734338; doi:10.3389/fpls.2020.580812)

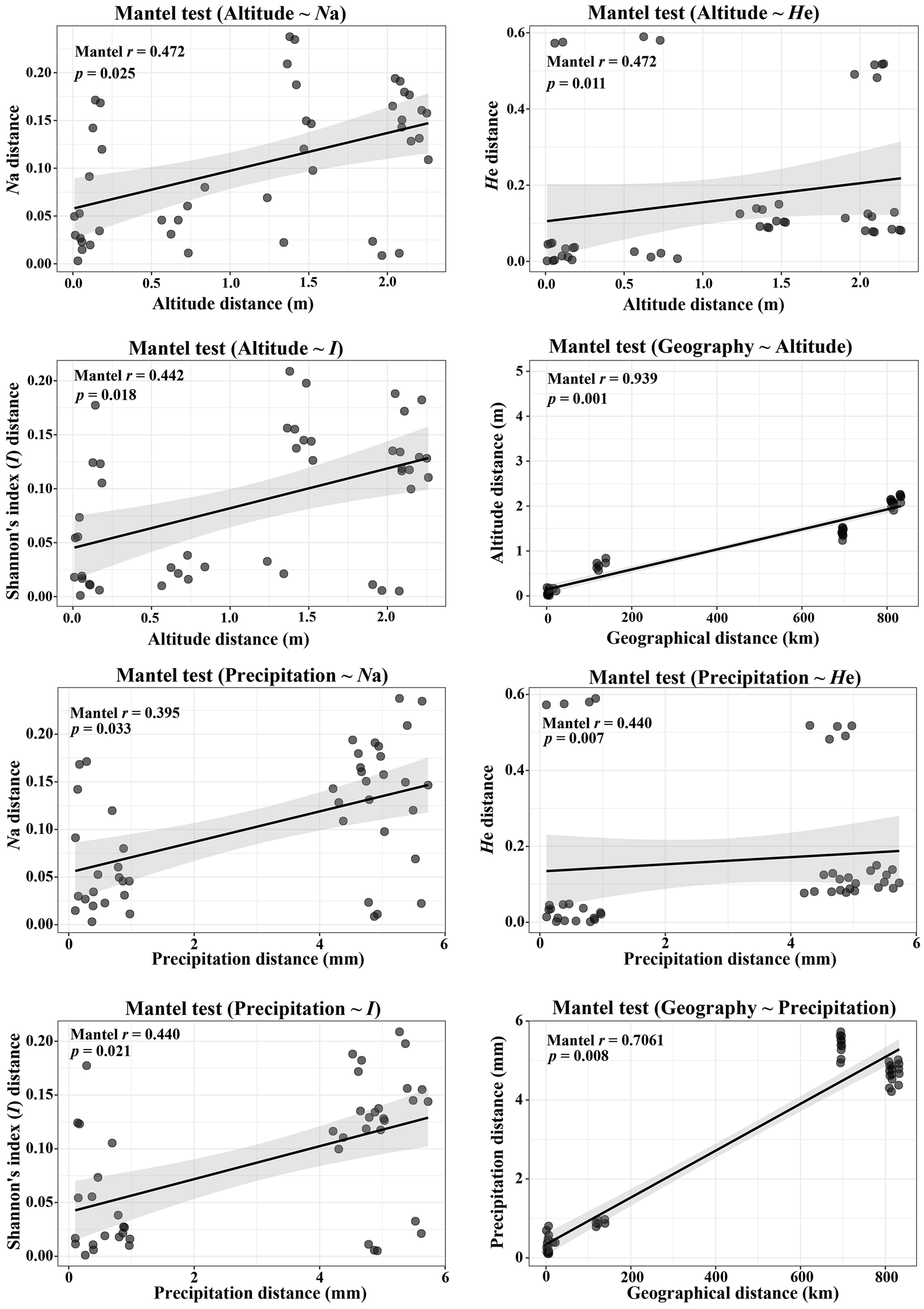

Supplement: Supplementary file 2 [file Image_1.TIF]

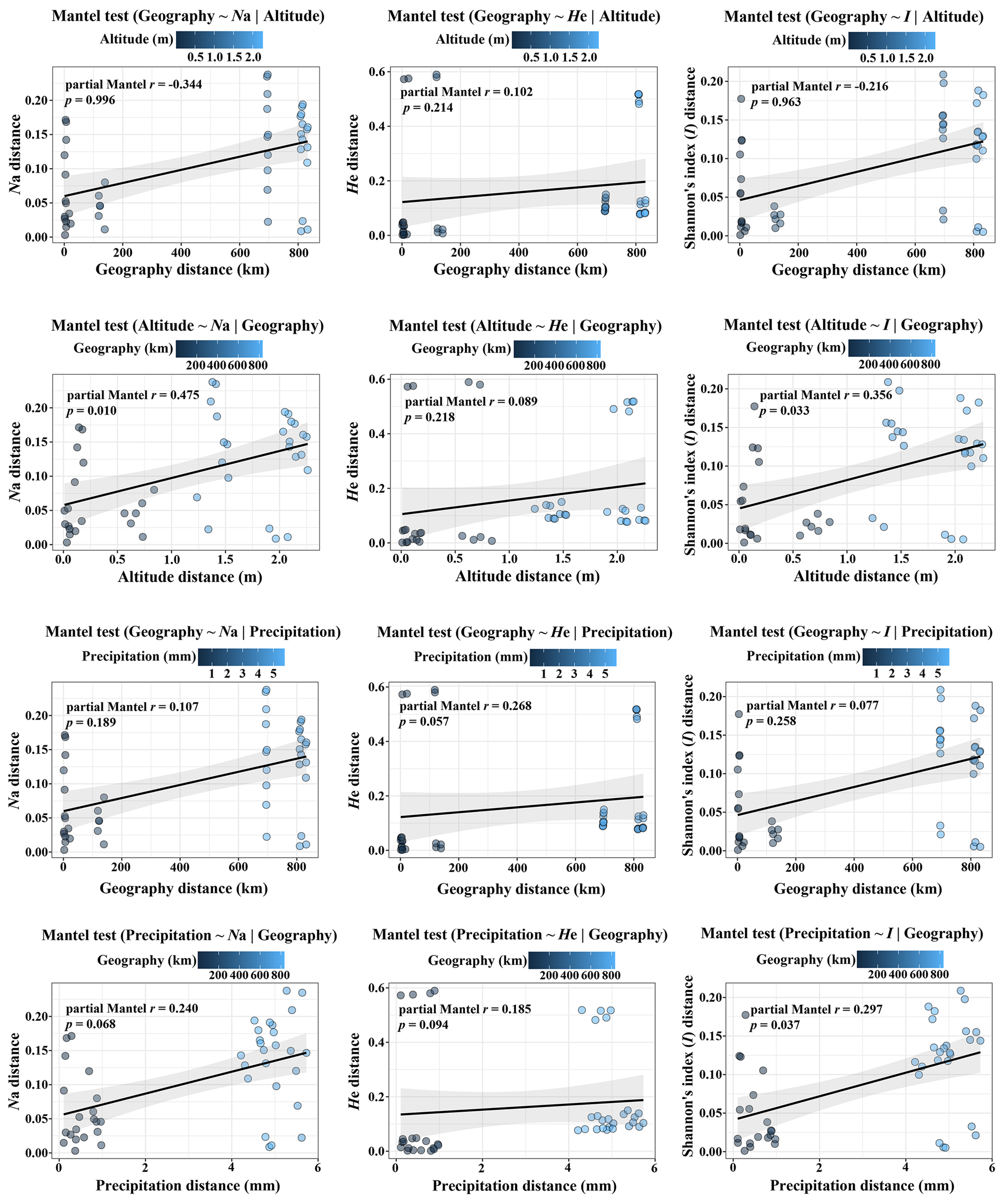

Supplement: Supplementary file 3 [file Image_2.TIF]

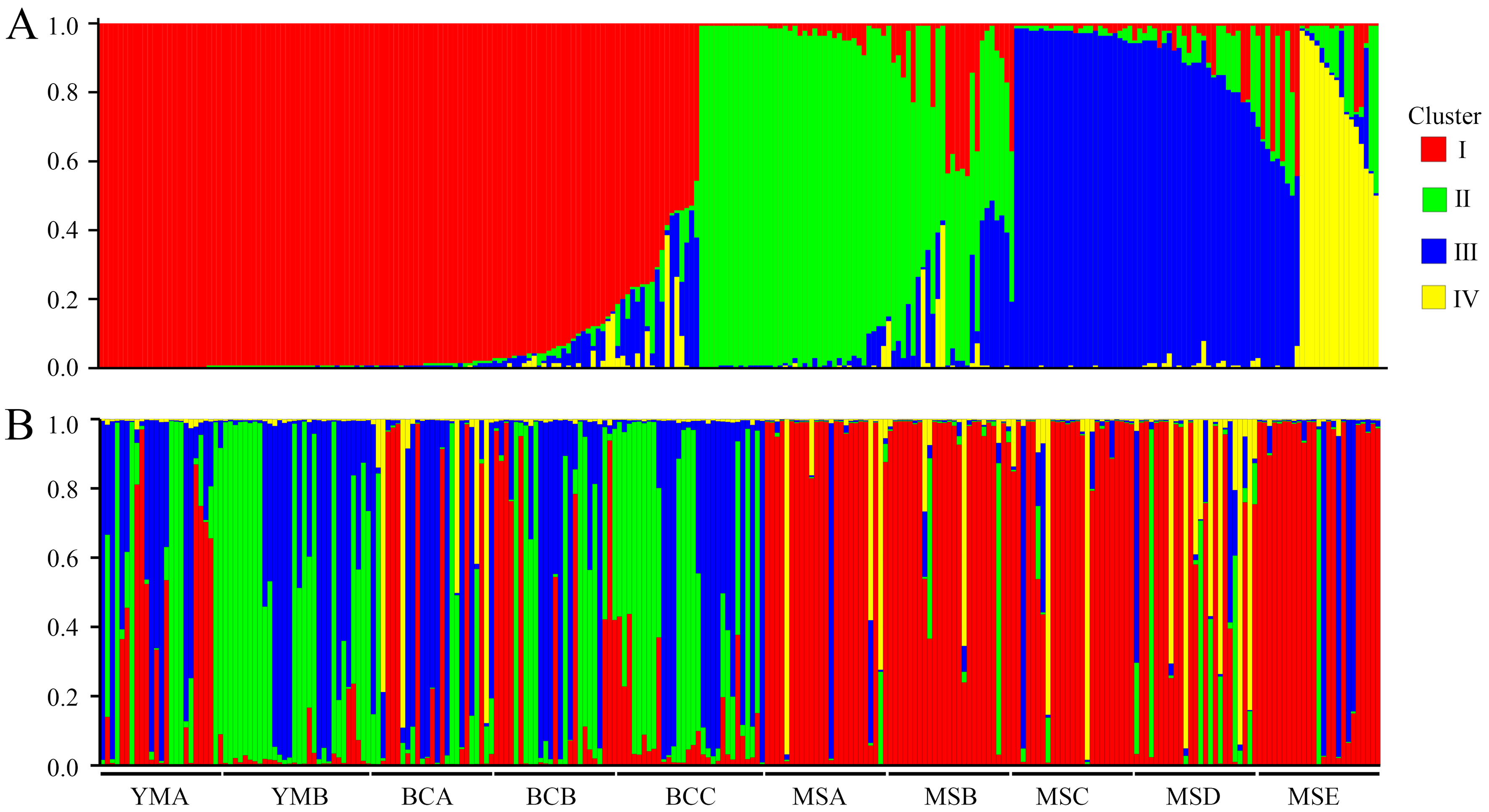

Supplement: Supplementary file 4 [file Image_3.TIF]

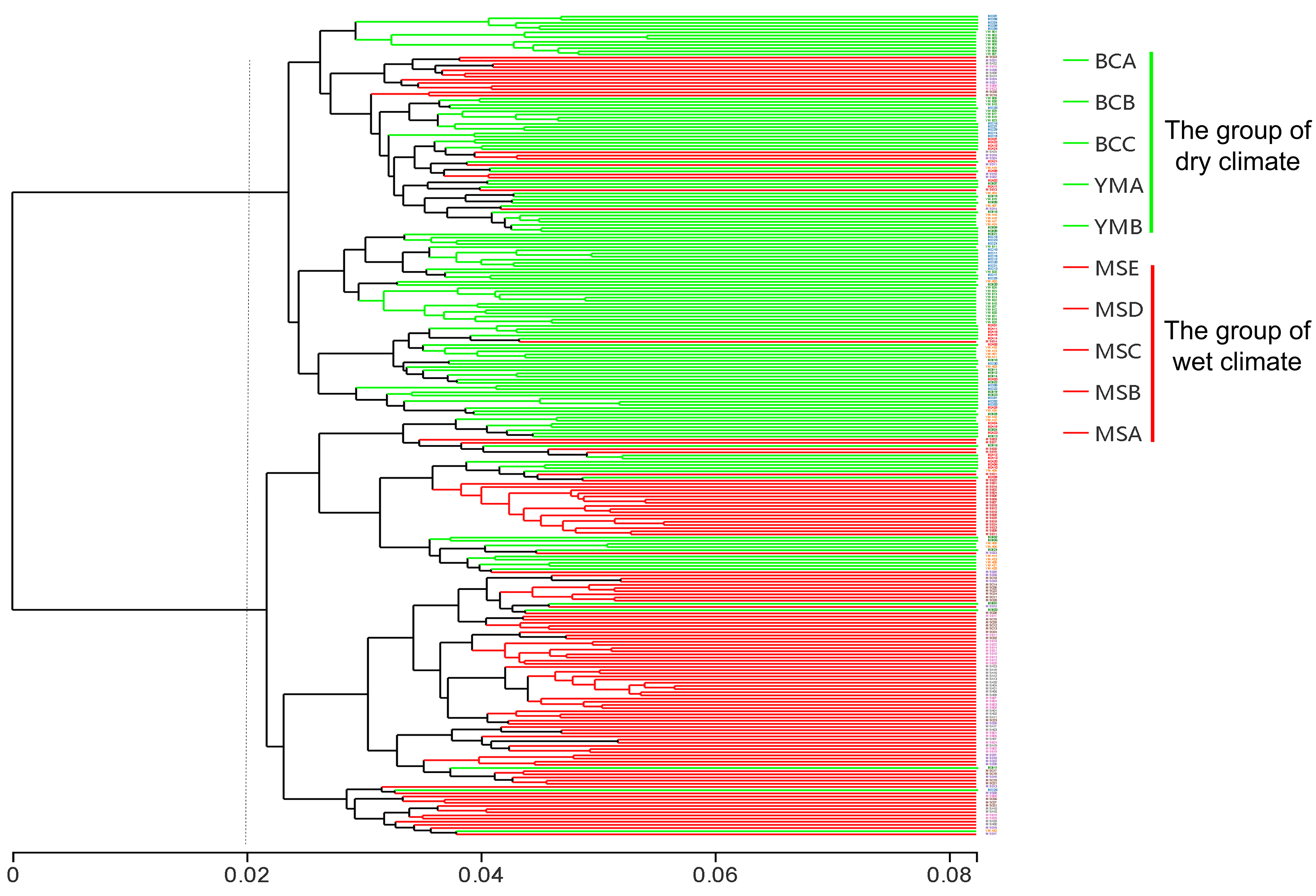

Supplement: Supplementary file 5 [file Image_4.TIF]

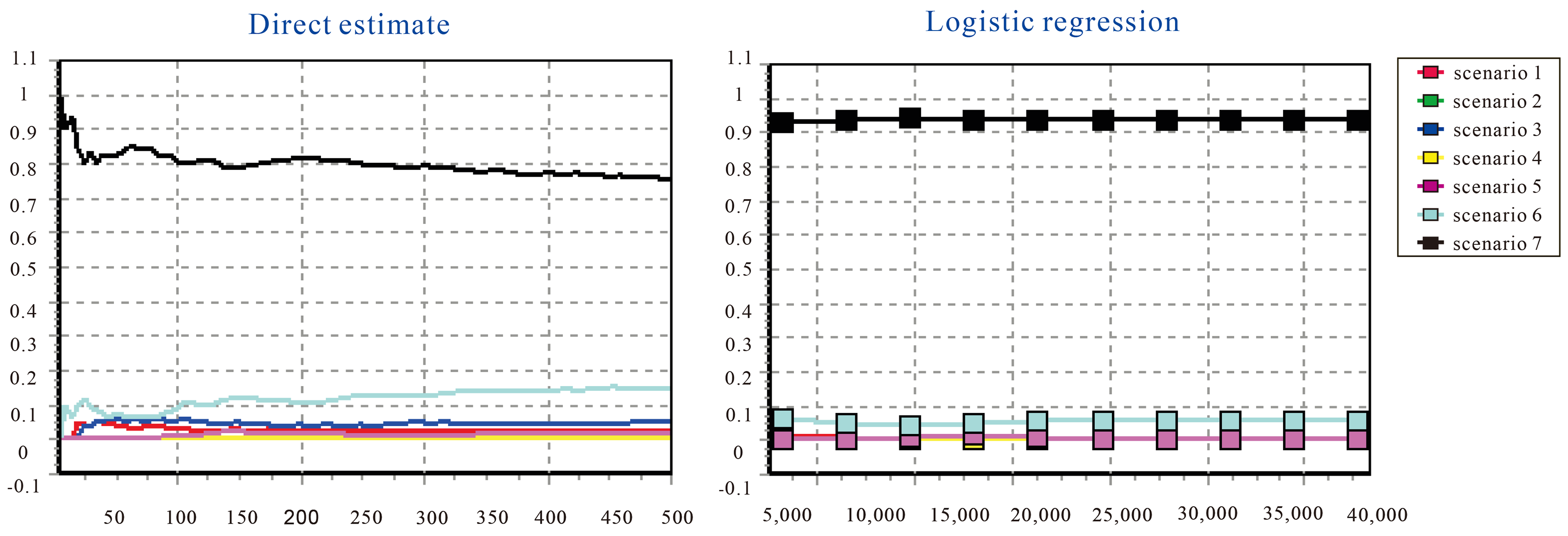

Supplement: Supplementary file 6 [file Image_5.TIF]
